# Exosomes: emerging biomarkers and therapeutic potential in postoperative delirium and postoperative cognitive dysfunction

**DOI:** 10.3389/fcell.2026.1854680

**Published:** 2026-08-18

**Authors:** Xinyu Li, Li Xu, Qiong Song, Qinjun Chu

**Affiliations:** 1 The Second Clinical Medical School of Zhengzhou University, Zhengzhou, Henan, China; 2 Department of Anesthesiology and Perioperative Medicine, Zhengzhou Central Hospital Affiliated to Zhengzhou University, Zhengzhou, China

**Keywords:** biomarkers, exosomes, neuroinflammation, postoperative cognitive dysfunction, postoperative delirium, targeted therapy

## Abstract

**Background:**

Postoperative delirium (POD) and postoperative cognitive dysfunction (POCD) are common and clinically significant perioperative neurocognitive disorders that disproportionately affect elderly surgical patients. Despite their substantial impact on postoperative recovery and long-term outcomes, the lack of validated biomarkers and effective targeted therapies remains a major challenge in perioperative medicine.

**Diagnostic Potential of Exosomes:**

Owing to their intrinsic ability to cross the blood-brain barrier and stably encapsulate central nervous system-derived biomolecules, exosomes have emerged as promising candidates for non-invasive biomarker discovery in POD and POCD. Disease-associated alterations in exosomal cargo, encompassing proteins such as P-tau and Aβ1-42, nucleic acids including miR-584–5p and circRNA_089763, and lipid-associated constituents, provide valuable molecular signatures for early diagnosis, disease stratification, prognostic assessment, and dynamic monitoring of perioperative neurocognitive disorders. It should be noted, however,that the majority of current evidence is extrapolated from Alzheimer’s disease and other neurodegenerative conditions rather than directly validated in POD and POCD cohorts, and dedicated prospective studies remain warranted.

**Therapeutic Applications of Exosomes:**

Exosomes exert therapeutic effects through two complementary mechanisms: serving as vehicles for targeted drug delivery and acting as endogenous neuroprotective mediators. Their excellent biocompatibility,low immunogenicity, and inherent capacity to traverse the blood-brain barrier render them attractive platforms for attenuating neuroinflammation, promoting synaptic repair, enhancing neurogenesis, and restoring blood-brain barrier integrity.

**Current Challenges and Future Perspectives:**

Despite their considerable promise, several challenges continue to impede clinical translation, including limitations in large-scale production, the lack of standardized isolation and characterization protocols, biological heterogeneity, and regulatory barriers. Future research should focus on establishing POD/POCD-specific exosomal biomarker panels, developing GMP-compliant manufacturing systems, engineering precision-targeted exosomal therapeutics, and conducting rigorous multicenter clinical studies to validate their safety and efficacy.

**Conclusion:**

Exosomes hold substantial promise for transforming the diagnosis and treatment of POD and POCD. With continued advances in engineering technologies, mechanistic understanding, and clinical validation, exosome-based diagnostic and therapeutic strategies are expected to become an integral component of precision perioperative medicine.

## 1 Introduction

With the global population aging and surgical techniques continuing to advance, the annual volume of major surgical procedures performed in elderly patients is steadily increasing. Postoperative neurocognitive complications, chiefly POD and POCD, have emerged as critical determinants of surgical outcomes and long-term patient health (Evered et al., 2018a). Postoperative delirium is characterized by acute, fluctuating disturbances in consciousness and cognitive function, and is closely associated with prolonged hospitalization, functional deterioration, and elevated mortality. Among elderly patients undergoing major procedures such as orthopedic or cardiac surgery, the reported incidence of POD reaches 23.6%, with substantially higher rates observed in specific high-risk subgroups, such as those undergoing aortic aneurysm repair (Gleason et al., 2015). POCD, in contrast, manifests as a persistent decline across multiple cognitive domains including memory, attention, and executive function, which may endure for months to years, significantly elevating the risk of long-term dementia and profoundly impairing patients’ quality of life (Moller et al., 1998).

At present, the diagnosis of perioperative cognitive impairment, encompassing both POD and POCD, lacks unified, standardized, and objective biological markers. Clinical assessment and non-standardized neuropsychological testing remain the primary diagnostic modalities, rendering cross-study comparisons difficult and impeding both mechanistic understanding and the development of effective interventions (Evered et al., 2018b). Although peripheral blood inflammatory markers and neuroimaging approaches have been investigated, their utility in preoperatively identifying high-risk individuals remains limited, as these measures tend to reflect pre-existing chronic neurodegenerative processes rather than specifically predicting acute postoperative cognitive decline. Their insufficient specificity and poor stability further constrain their applicability as early-warning tools in clinical practice (Crosby et al., 2011). Accordingly, the identification of specific biomarkers and the development of innovative therapeutic strategies remain among the foremost challenges in perioperative medicine.

Exosomes are extracellular vesicles ranging from 30 to 150 nm in diameter, ubiquitously present in biofluids including blood, cerebrospinal fluid, and urine (Welsh et al., 2024). Enclosed within a lipid bilayer membrane, these vesicles stably encapsulate a diverse repertoire of bioactive molecules derived from their parent cells, including proteins, microRNAs (miRNAs), mRNAs, and lipids, and facilitate intercellular communication by delivering this molecular cargo to recipient cells (Valadi et al., 2007). A defining advantage of exosomes as diagnostic markers and therapeutic carriers lies in their inherent capacity to traverse the blood-brain barrier. Preclinical studies have demonstrated that engineered targeted exosomes administered via systemic injection can effectively deliver therapeutic nucleic acids such as siRNA to specific neuronal populations within the brain, providing critical proof of principle for harnessing exosomes to mediate directed molecular exchange between the central nervous system and the periphery (Alvarez-Er et al., 2011). Recent investigations have further shown that neuron-derived brain extracellular vesicles isolated from peripheral blood carry molecular constituents including Aβ, phosphorylated tau, and α-synuclein that faithfully reflect neuropathological changes in patients with Alzheimer’s and Parkinson’s disease (Fiandaca et al., 2015; Goetzl et al., 2015). Crucially, multiple studies have confirmed that the diagnostic performance of blood-based NDE biomarkers is highly concordant with and comparable to that of conventional cerebrospinal fluid analysis (Jia et al., 2019; Shi et al., 2014), offering new minimally invasive tools for the diagnosis and longitudinal monitoring of neurodegenerative disease. A growing body of evidence indicates that the pathophysiological mechanisms underlying POD and POCD substantially overlap with those of chronic neurodegenerative conditions such as Alzheimer’s disease (Safavynia and Goldstein, 2019). Shared core pathways include persistent neuroinflammation precipitated by surgical stress, consequent synaptic dysfunction and loss, neuronal apoptosis, and a self-reinforcing cycle driven by blood-brain barrier disruption. This mechanistic convergence implicates exosomes as potentially pivotal mediators in the pathogenesis of these postoperative complications. Emerging research suggests that exosomes may function as critical intermediaries linking peripheral surgical stress to central nervous system injury. Surgical trauma, anesthetic exposure, and perioperative stress responses activate peripheral immune cells, triggering a surge of pro-inflammatory cytokines into the systemic circulation, during which various cell types release exosomes in substantial quantities. Stressed and apoptotic neurons in particular alter the molecular composition of their released exosomes, favoring the packaging of injury-signaling molecules. Concurrently, surgical insult compromises tight junction proteins within the blood-brain barrier, facilitating the infiltration of peripheral factors into the brain parenchyma. These circulating exosomes, upon breach of blood-brain barrier integrity, penetrate the central nervous system and are taken up by microglia, astrocytes, and neurons. Through the delivery of pro-inflammatory cytokines, aberrant miRNAs, or neurotoxic proteins, they drive microglial activation, synaptic dysfunction, neuronal injury, and sustained neuroinflammation. This persistent neuroinflammatory milieu further disrupts hippocampal neurogenesis and synaptic plasticity, ultimately manifesting clinically as POD or POCD. Exosomes may thus function as messengers that propagate peripheral surgical stress signals to the central nervous system, potentially contributing to the pathological progression of these conditions, though this mechanistic framework requires direct validation in POD and POCD models. This mechanistic role constitutes the fundamental rationale for their identification in the present review as emerging biomarkers for the early diagnosis of POD and POCD. Beyond their pathogenic role, exosomes transport brain-derived pathological molecules into the peripheral circulation, providing a non-invasive window into CNS-specific pathology. Simultaneously, they may serve as natural drug delivery vehicles or exert direct neuroprotective effects through their endogenous cargo (Fiandaca et al., 2015; Shi et al., 2014), with certain reparative exosome populations such as those derived from stem cells directly modulating neuroinflammation and synaptic plasticity via cargo molecules including miRNAs (Doeppner et al., 2015).

In summary, this review advances and substantiates the central thesis that, by virtue of their unique bidirectional brain-periphery communication capacity, exosomes offer an integrated closed-loop solution for POD and POCD management that encompasses early diagnosis, precision intervention, and dynamic monitoring. This review synthesizes the latest research advances pertaining to exosomes in the context of POD and POCD, comprehensively explores their potential as both biomarkers and therapeutic tools, critically analyzes the major challenges currently confronting the field, and charts future directions for accelerating their translation into clinical practice.

## 2 Exosomes as potential biomarkers for POD and POCD

**FIGURE 1:**
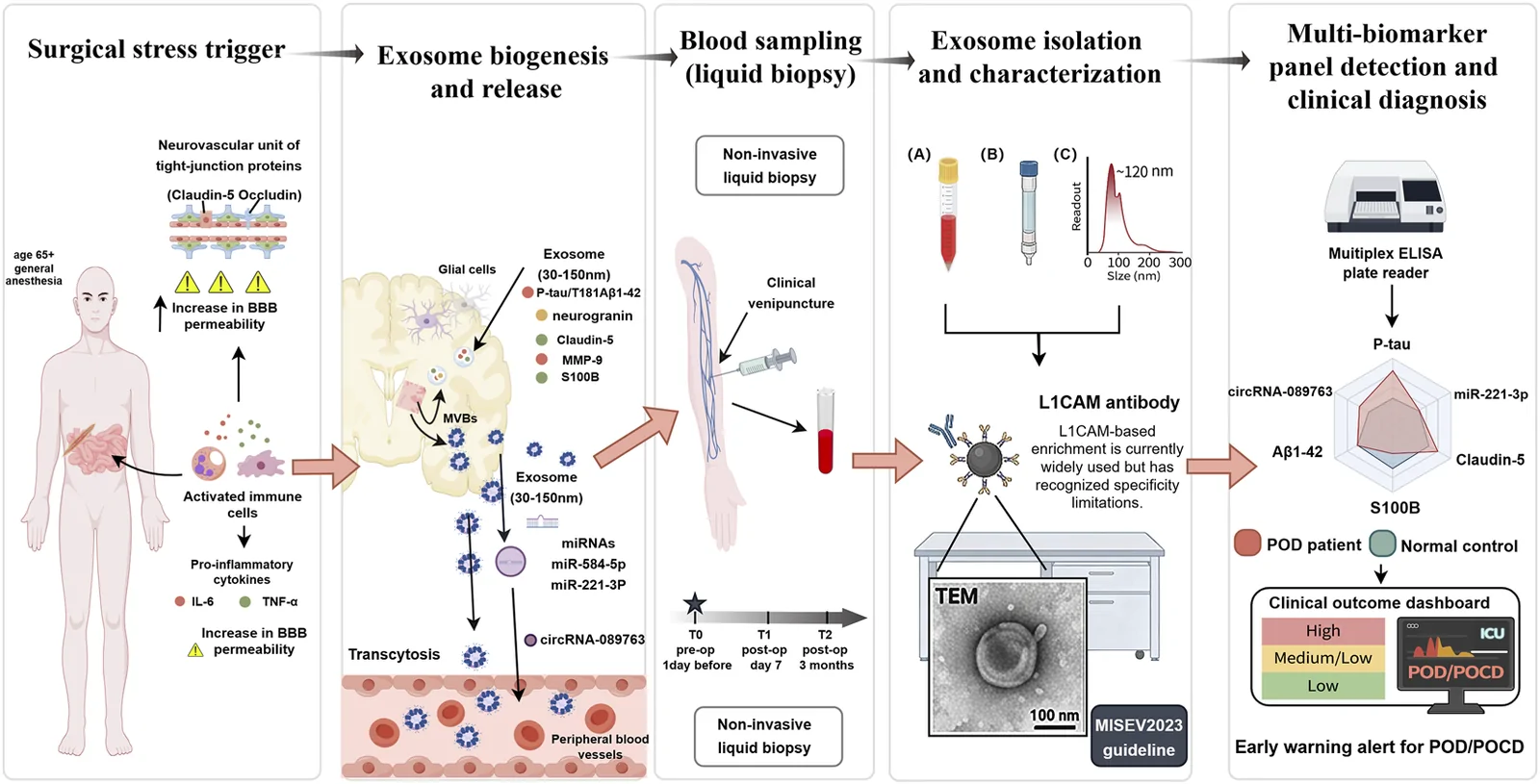
A complete exosome-based biomarker workflow for the diagnosis of POD and POCD. Surgical stress in elderly patients undergoing general anaesthesia triggers peripheral immune cell activation and increased BBB permeability, promoting exosome biogenesis and release from glial cells into peripheral blood vessels via transcytosis. Exosomes (30–150 nm) carry candidate biomarkers including P-tau/T181Aβ1-42, neurogranin, Claudin-5, MMP-9, and S100B as protein cargo, and miR-584–5p, miR-221–3p, and circRNA-089763 as nucleic acid cargo. Following minimally invasive peripheral venipuncture at three time points (T0:pre-op 1 day before; T1:post-op day 7; T2:post-op 3 months), exosomes are isolated and characterized using L1CAM antibody-based magnetic bead pulldown to enrich neuron-derived exosomes (note:L1CAM-based enrichment is currently widely used but has recognized specificity limitations), with vesicle size and morphology confirmed by nanoparticle tracking analysis (∼120 nm peak) and transmission electron microscopy (TEM), in accordance with MISEV2023 guidelines. A multi-biomarker panel comprising P-tau, Aβ1-42, Claudin-5, S100B, miR-221–3p, and circRNA-089763 is subsequently quantified using a multiplex ELISA plate reader and bioinformatics AI algorithms, generating a radar plot to discriminate POD patients from normal controls, and a clinical outcome dashboard providing early warning stratification (High/Medium/Low risk) for POD/POCD. Abbreviations:BBB, blood-brain barrier; MVBs, multivesicular bodies; TEM, transmission electron microscopy; MISEV, Minimal Information for Studies of Extracellular Vesicles; ICU, intensive care unit. Created with Figdraw.

The primary advantage of exosomes as biomarkers resides in their capacity to selectively encapsulate molecules of central nervous system origin and maintain stability within the peripheral circulation, thereby circumventing the well-recognized limitations of conventional biomarkers, including poor specificity and susceptibility to proteolytic degradation (Fiandaca et al., 2015; Shi et al., 2014). In the context of the pathological processes underlying POD and POCD, such as neuronal injury and neuroinflammation, the molecular composition of exosomal cargo undergoes characteristic alterations. These disease-associated molecular signatures thus hold considerable promise as biomarkers for the diagnosis, disease staging, and prognostic assessment of perioperative neurocognitive disorders. The overall workflow integrating exosome biogenesis, isolation, biomarker detection, and clinical diagnosis is illustrated in Figure 1. It should be noted that the evidence reviewed in this section spans two distinct levels:direct evidence validated in human POD and POCD cohorts, and analogous evidence extrapolated from Alzheimer’s disease and related neurodegenerative conditions on the basis of shared pathological mechanisms; the latter is explicitly identified throughout and should be interpreted with appropriate caution.

### 2.1 Protein biomarkers in exosomes

Exosomal proteins currently represent one of the most extensively investigated categories of exosome-based biomarkers. It is worth noting that members of the tau protein family and Aβ-related proteins are well-established biomarkers in the broader context of neurodegenerative disease research; however, the present discussion focuses specifically on these molecules as cargo constituents of neuron-derived exosomes, alongside synaptic proteins. By virtue of the stability and cell-type traceability conferred by the exosomal compartment, these molecularly characterized cargo species are regarded as particularly promising candidates for exosome-specific biomarker development in perioperative neurocognitive disorders.

Phosphorylated tau (P-tau), particularly the P-T181 and P-S396 isoforms, serves as a definitive marker of neuronal injury (Blennow and Hampel, 2003). In the context of Alzheimer’s disease research, levels of these P-tau subtypes in cerebrospinal fluid (CSF)-derived exosomes are significantly elevated relative to controls, with diagnostic accuracy comparable to that of conventional CSF-based detection methods (Fiandaca et al., 2015). Evidence from basic research further indicates that perioperative stressors, including anesthetic exposure, surgical trauma, and the ensuing systemic inflammatory response, can directly perturb central nervous system homeostasis. These insults activate key tau kinases and induce persistent neuroinflammation, thereby jointly promoting aberrant tau phosphorylation and pathological intraneuronal accumulation (Planel et al., 2009; Terrando et al., 2011). The progressive accrual of hyperphosphorylated tau ultimately precipitates severe impairment of neuronal function, synaptic loss, and neuronal death, culminating in the formation of neurofibrillary tangles (NFTs), a cardinal neuropathological hallmark of Alzheimer’s disease. Although existing evidence does not establish exosomal tau proteins as specific biomarkers of POD or POCD, the pathological alterations described above share mechanistic commonalities with the core neuropathological processes underlying these conditions. It is therefore reasonable to postulate that aberrant tau phosphorylation and its exosomal dissemination may contribute, at least in part, to the molecular basis of perioperative cognitive impairment (Needham et al., 2017).

Amyloid-β42 (Aβ1-42)constitutes another key biomarker of relevance in this context. A substantial body of basic research evidence indicates that perioperative stressors, including anesthetic exposure and surgical trauma, can disrupt the metabolic equilibrium of amyloid-β in the brain. Inhalation anesthetics at clinically relevant concentrations, such as isoflurane, have been shown to significantly augment Aβ production and promote its accumulation in both cellular and transgenic mouse models (Xie et al., 2006). Aβ accumulation has been shown to exert synaptic toxicity, impairing long-term potentiation and precipitating the loss of dendritic spines in experimental models (Shankar et al., 2008). These processes are proposed to represent important upstream contributors to synaptic dysfunction in the setting of postoperative cognitive impairment, though direct causal evidence in POD/POCD specifically remains to be established. Plasma Aβ1-42 serves as a representative example of this biomarker class. Prior studies have suggested that elevated Aβ1-42 levels may impair hippocampal cognitive and metabolic function by disrupting region-specific insulin signaling pathways, a mechanism that may be relevant to the neuronal dysfunction observed in the context of postoperative cognitive impairment (Pearson-Leary and McNay, 2012). Jia et al. reported that Aβ1-42 levels in plasma neuron-derived exosomes from patients with Alzheimer’s disease exhibited a significant positive correlation with corresponding concentrations measured in cerebrospinal fluid (Jia et al., 2019). These findings suggest that aberrant Aβ1-42 levels in plasma-derived exosomes may serve as a peripheral reflection of dysregulated intracerebral amyloid-β metabolism. Relative to the measurement of free plasma Aβ42, the fundamental advantage of detecting Aβ1-42 within plasma-derived exosomes lies in the protective function of the exosomal lipid bilayer, which effectively shields its encapsulated cargo from proteolytic hydrolysis and degradation in the systemic circulation, thereby enabling more accurate and reliable quantification (Wu et al., 2022). Although direct evidence linking exosomal Aβ1-42 specifically to POD or POCD remains lacking, emerging studies have begun to implicate neurodegenerative biomarkers, including amyloid-β42, as potential predictors of POCD. Rigorous prospective clinical trials will nonetheless be required to validate this hypothesis and establish the clinical utility of exosomal Aβ1-42 in the perioperative neurocognitive setting.

Synaptic proteins play an indispensable role in maintaining normal cognitive function. Recent studies have demonstrated that neuron-derived exosomes released into the peripheral circulation stably encapsulate a diverse array of synaptic proteins, rendering them accessible for minimally invasive detection in peripheral blood (Nakamura et al., 2018). Evidence suggests that levels of synaptic proteins, including synaptophysin and neurogranin, encapsulated within neuron-derived exosomes are already diminished in patients with frontotemporal dementia prior to symptom onset (Goetzl et al., 2018). Furthermore, this evidence also reported that reduced levels of excitatory synaptic proteins, such as NPTX2,NRXN2α, AMPA4, and NLGN1, in NDEs have been associated with cognitive impairment in Alzheimer’s disease. Although this condition is distinct from POD and POCD, its underlying pathological mechanisms converge on synaptic dysfunction as a shared core feature. Dedicated prospective clinical studies are therefore warranted to systematically characterize the expression profiles of synapse-related proteins in exosomes derived from POD and POCD patients, and to determine whether such profiles may serve as viable preclinical monitoring indicators for these perioperative neurocognitive disorders.

Beyond synaptic proteins, inflammation-related proteins carried within exosomes demonstrate considerable diagnostic potential. Postoperative neuroinflammation is well recognized as a key pathological driver of both POD and POCD (Terrando et al., 2011). Conventional inflammatory markers such as IL-6, CRP, and S-100β have been supported by numerous studies as indicators of perioperative neuroinflammation, yet research into their exosome-encapsulated counterparts as specific biomarkers for POD and POCD remains at an early and evolving stage (Fauré et al., 2006; Goetzl et al., 2016; Zhang et al., 2024). In this regard, Yang et al. demonstrated that C8G, one of the three subunits of complement component 8 and a protein primarily upregulated in the inflamed brains of patients with Alzheimer’s disease, was significantly elevated in astrocyte-derived exosomes and showed significant correlations with global cognitive scores as well as performance across all cognitive subdomains. These findings collectively indicate that exosomal complement proteins can faithfully reflect the severity of cerebral inflammation, underscoring their potential as sensitive reporters of neuroinflammatory burden and as a novel diagnostic avenue for perioperative neurocognitive disorders (Yang et al., 2023). Notably, the shared pathological basis between POD/POCD and Alzheimer’s disease may reside in an acute neuroinflammatory response triggered by surgical stress, characterized by the activation of astrocytes and microglia (Terrando et al., 2011). On this basis, longitudinal monitoring of inflammation-related proteins in serum-derived exosomes represents a promising and clinically tractable approach for the early prevention and risk stratification of POD and POCD.

### 2.2 Nucleic acid biomarkers in exosomes

Exosomes serve as important mediators of intercellular communication, stably encapsulating within their lumen a diverse repertoire of bioactive nucleic acids, including miRNAs, lncRNAs, and circRNAs (Valadi et al., 2007). Upon delivery to recipient cells, these nucleic acid species participate in the regulation of gene expression at both the post-transcriptional and transcriptional levels (Valadi et al., 2007; Statello et al., 2021). Studies have demonstrated that neuron-derived exosomes contribute to the regulation of synaptic plasticity and neuronal homeostasis through this mechanism (Goldie et al., 2014). The role of miRNAs in POD and POCD is multifaceted, as they operate within complex regulatory networks that synergistically modulate key pathological processes including neuroinflammation, neuronal apoptosis, and oxidative stress. Studies have shown that hippocampal neurons exposed to sevoflurane anesthesia secrete exosomes enriched with miR-584–5p, which are subsequently taken up by microglia. This intercellular transfer was associated with suppression of microglial BDNF expression, synaptic damage, and microglial dysfunction *in vitro*, suggesting a potential mechanistic link to the development of POCD. With respect to POD specifically, no studies have yet established a direct link between exosomal miRNAs and this condition; however, instructive insights can be drawn from parallel research in other neurodegenerative diseases. Prior studies have reported that exosomal miR-146a, and miR-125 b in peripheral biofluids such as blood are associated with the early diagnosis, disease classification (Dong et al., 2015; Duan et al., 2024), and longitudinal monitoring of Alzheimer’s disease. Similarly, exosomal miR-223–3p, miR-101–3p, and related species in plasma have been found to be closely associated with cerebrovascular disease and cognitive impairment (Zhao et al., 2021; Zhang H. et al., 2025). Given the well-documented mechanistic commonalities between POD/POCD and these neurodegenerative conditions, these findings carry significant translational potential and warrant dedicated investigation in the perioperative neurocognitive context. Long non-coding RNAs(lncRNAs) represent important epigenetic regulatory factors with broad relevance to neurological disease. Review analyses have indicated that lncRNAs such as BACE1-AS in Alzheimer’s disease and UCHL1-AS in Parkinson’s disease directly participate in the regulation of core pathological pathways, including Aβ generation and α-synuclein metabolism, either by directing chromatin modification complexes to specific genomic loci or by stabilizing pathogenic protein-encoding mRNAs (Zhao et al., 2022). These findings provide a mechanistic framework for interpreting the potential functional consequences of disease-associated alterations in exosomal lncRNA composition. The clinical diagnostic value of exosomal lncRNAs has already been established across multiple disease contexts. For instance, the combined detection of FOXD1-AS1, NRIR, and XLOC009459 has been shown to reflect colorectal cancer tumor burden in real time (Yu, 2022), while H19 and MIAT have been identified as biomarkers for gastric cancer (Deng et al., 2023), and GASS, MALAT-1, and SOX2-OT have demonstrated diagnostic potential in lung cancer (Huang and Hu, 2023). With respect to cognitive dysfunction, although no studies have yet directly established a relationship between lncRNAs and POD or POCD, preliminary evidence has emerged from diabetic cognitive dysfunction models. In this context, serum exosomal lncRNA MALAT1 has been proposed to traverse the blood-brain barrier and modulate miR-382–3p in the hippocampus, thereby enhancing the expression of brain-derived neurotrophic factor (BDNF) and ameliorating cognitive deficits in mice (Wang et al., 2023). Research at the intersection of POD/POCD and exosomal lncRNAs remains in its exploratory phase, and no directly relevant clinical studies have yet been reported. Nevertheless, the methodological frameworks established in exosomal miRNA research in POCD may serve as a productive foundation from which lncRNA-focused investigations can be developed and extended.

Exosome-encapsulated circRNAs, by virtue of their exceptional structural stability, are particularly well suited as non-invasive diagnostic or prognostic biomarkers. In addition to their biomarker potential, circRNAs can function as miRNA sponges, thereby regulating target genes implicated in neuroinflammation and synaptic plasticity. Differentially expressed circRNAs have already been identified in exosomes derived from POD and POCD patients (Wang et al., 2019). One such study confirmed the potential of circRNA089763 as a POCD biomarker and, for the first time, established a link between exosome-bound circRNA and neurocognitive complications following coronary artery bypass grafting (CABG). The study further proposed that circRNA may contribute to the pathogenesis of POCD through a miRNA sponge mechanism, indirectly influencing cognitive function by modulating the expression of IGFBP5, STC, and YWHAG. A summary of the key studies on candidate exosomal biomarkers discussed above is presented in Table 1.

**TABLE 1:** Research on associated biomarkers.

Candidate biomarker	P-T181-tau/P-S393-tau (Jia et al., 2019)	Aβ1-42 (Jia et al., 2019)	Synaptic proteins (Goetzl et al., 2018)	C8G (Yang et al., 2023)	miR-584–5p (Zhao et al., 2022)	circRNA 089763 (Wang et al., 2019)
Exosome source	Plasma neuron-derived extracellular vesicles (NDEVs)	Plasma neuron-derived exosomes (NDEs)	Plasma neuron-derived exosomes (NDEs)	Plasma astrocyte-derived exosomes (ADEs)	Plasma exosomes	Plasma exosomes
Study design	Prospective RCT	Cross-sectional case-control	Cross-sectional + longitudinal case-control	Cross-sectional cohort	Prospective cohort	Prospective cohort
Sample size	116 mild-to-moderate AD patients 20 healthy controls	Discovery:AD 43, aMCI 25, Controls 29 Validation:AD 73, aMCI 71, Controls 72	Cross-sectional:AD 12, FTD16, controls 28; Longitudinal:AD 9, FTD 10, controls 19	124 OSA patients with MCI 100 OSA patients without MCI 50 healthy controls	10 POCD patients 10 healthy controls	82 patients undergoing CABG
Key findings	P-T181-tau and P-S393-tau levels in plasma NDEVs were significantly elevated in AD patients relative to controls; levels did not increase further with disease severity, suggesting that changes are most prominent at early or specific disease stages	Plasma NDE Aβ1-42 levels were significantly elevated in AD patients and showed a significant positive correlation with corresponding CSF levels	Levels of synaptophysin, synaptopodin,synaptotagmin-2,and neurogranin in NDEs were significantly lower in AD and FTD patients than in healthy controls; synaptotagmin, synaptophysin,and neurogranin declined significantly years before the onset of overt dementia symptoms, suggesting early-warning potential	C8G concentrations in ADEs were significantly elevated in OSA patients with MCI and showed an independent positive correlation with cognitive impairment and increased white matter hyperintensity volume	miR-584–5p levels were significantly elevated in plasma exosomes of sevoflurane-induced POCD patients; *in vitro* experiments showed miR-584–5p markedly suppressed HMC3 cell viability, promoted apoptosis,and upregulated pro-inflammatory cytokines IL-1β and TNF-α	circRNA_089763 was significantly upregulated in the POCD group and was the only stably elevated marker; bioinformatic analysis identified putative target genes enriched in POCD-related GO terms and signalling pathways, including IGFBP5,STC,and YWHAG.
Diagnostic performance	Not reported	Not reported	Not reported	Not reported	Not reported	Not reported
Main limitations	Small sample size; limited observation window	The causal relationship between the identified markers and POD/POCD has not been established,no comparative analysis against existing diagnostic modalities was performed, and the statistical power of the study may be insufficient.	Small sample sizes; predominantly cross-sectional design	Inherent limitations of cross-sectional design; limited external validity	Based on *in vitro* cell experiments only, lacking *in vivo* animal model validation; very small sample size; single aetiological model	Mechanistic investigation limited to bioinformatic prediction; no in-depth correlation with POCD severity; short follow-up duration
Relevance to POD/POCD	Aberrant tau phosphorylation and pathological accumulation are associated with neuronal dysfunction in neurodegenerative disease contexts, overlapping with proposed core pathological mechanisms of POD/POCD.	Aβ accumulation has been shown to exert synaptic toxicity and impair long-term potentiation in experimental models, processes proposed as potential upstream contributors to synaptic dysfunction in postoperative cognitive impairment.	Abnormal synaptic protein levels are associated with alterations in synaptic structure and function, consistent with proposed pathological mechanisms of cognitive dysfunction in POD/POCD.	C8G is an inflammatory mediator involved in neuroinflammatory processes and is significantly correlated with global cognitive function, offering a potential index of neuroinflammatory burden in POD/POCD.	Exosomes from sevoflurane-induced POCD patients were found to carry elevated miR-584–5p,which was associated with suppression of BDNF expression and microglial dysfunction *in vitro*, suggesting a potential mechanistic role in POCD pathogenesis that warrants further *in vivo* validation.	Exosomal circRNAs participate in neuroinflammation and cognitive dysfunction through post-transcriptional regulatory networks via a miRNA sponge mechanism, with direct relevance to POCD.

### 2.3 Advantages of exosome biomarkers

Relative to conventional biomarkers, exosome-based biomarkers confer several distinctive advantages in the diagnosis of POD and POCD.

In terms of molecular richness and specificity, exosomes carry a source-cell-specific and rigorously sorted cargo of bioactive molecules, encompassing proteins, nucleic acids including miRNA, mRNA,and lncRNA, as well as lipids and metabolites. This multidimensional, high-information-density molecular repertoire enables a more comprehensive reflection of the physiological and pathological state of the originating cell. Neuron-derived brain extracellular vesicles in particular originate from neural cells, and their cargo specifically mirrors pathological alterations within the central nervous system, thereby minimizing confounding contributions from peripheral tissues (Fiandaca et al., 2015).

With regard to accessibility and widespread distribution, exosomes are present across a broad range of biological fluids, including plasma, urine, and cerebrospinal fluid, and their collection is inherently non-invasive. Peripheral blood sampling is simple and routinely feasible, representing a considerable practical advantage over the invasive procedure of cerebrospinal fluid collection, and providing a readily accessible source for repeated and dynamic clinical monitoring. As key mediators of intercellular communication, exosomes are capable of reflecting pathological changes in the central nervous system. Studies have demonstrated that exosomes can yield diagnostically informative signatures across multiple subtypes of dementia, offering new perspectives for the diagnosis of cognitive disorders including POD and POCD (Joo et al., 2023). Notably, the capacity of exosomes to traverse the blood-brain barrier and transport brain-specific biomarkers into the peripheral circulation positions them as an ideal non-invasive diagnostic tool.

Concerning stability and cargo protection, the lipid bilayer membrane structure of exosomes constitutes a natural physical barrier for their encapsulated bioactive molecules, enabling effective resistance to enzymatic degradation during circulation in biological fluids as well as during sample storage. This confers a level of molecular stability that ensures the integrity and reliability of downstream analytical results (Kalluri and LeBleu, 2020). Yuan et al. demonstrated that the exosomal lipid bilayer effectively protects encapsulated RNA, such as DNACR, from degradation by RNase A (Yuan et al., 2019). Complementary evidence was provided by Wang et al., who showed that upon disruption of the exosomal lipid membrane with the surfactant NP-40, the internal RNA became susceptible to RNase A-mediated degradation, offering reverse validation of the protective function conferred by membrane integrity (Wang et al., 2016). Beyond the physical barrier provided by the lipid bilayer itself, the stability of exosomal cargo may be further reinforced through biochemical mechanisms. Evidence suggests that RNA and proteins encapsulated within exosomes can form stable complexes with specific proteins and glycans present on the exosomal membrane surface, an interaction that contributes independently to the preservation of molecular integrity during circulation and storage (Sta et al., 2018; Sharma et al., 2025).

With respect to disease association and early diagnostic potential, the surface protein profile and molecular cargo composition of exosomes are both cell-type-specific and state-dependent. Under pathological conditions, these molecular constituents undergo characteristic alterations, enabling exosomes to function as disease-specific molecular fingerprints applicable to conditions ranging from specific tumor subtypes to neurodegenerative disorders, and conferring upon them the potential to serve as highly specific diagnostic biomarkers (Mathivanan et al., 2010; Hoshino et al., 2020). Studies have demonstrated that exosomes derived from lung adenocarcinoma highly express the surface protein EGFR, whereas those originating from lung squamous cell carcinoma specifically express CD151, illustrating the precision with which exosomal surface profiles can discriminate between disease subtypes. Importantly, exosomal biomarker alterations can precede the emergence of overt clinical symptoms, thereby creating a window for early intervention and potentially improving patient outcomes (Clark et al., 2016). This is further exemplified by findings in non-small cell lung cancer, where an increase in the abundance of the drug resistance-associated T790 M mutation has been detected in circulating exosomes prior to imaging evidence of tumor progression (Castellanos-Rizaldos et al., 2018).

## 3 Therapeutic potential of exosomes in POD and POCD

**FIGURE 2:**
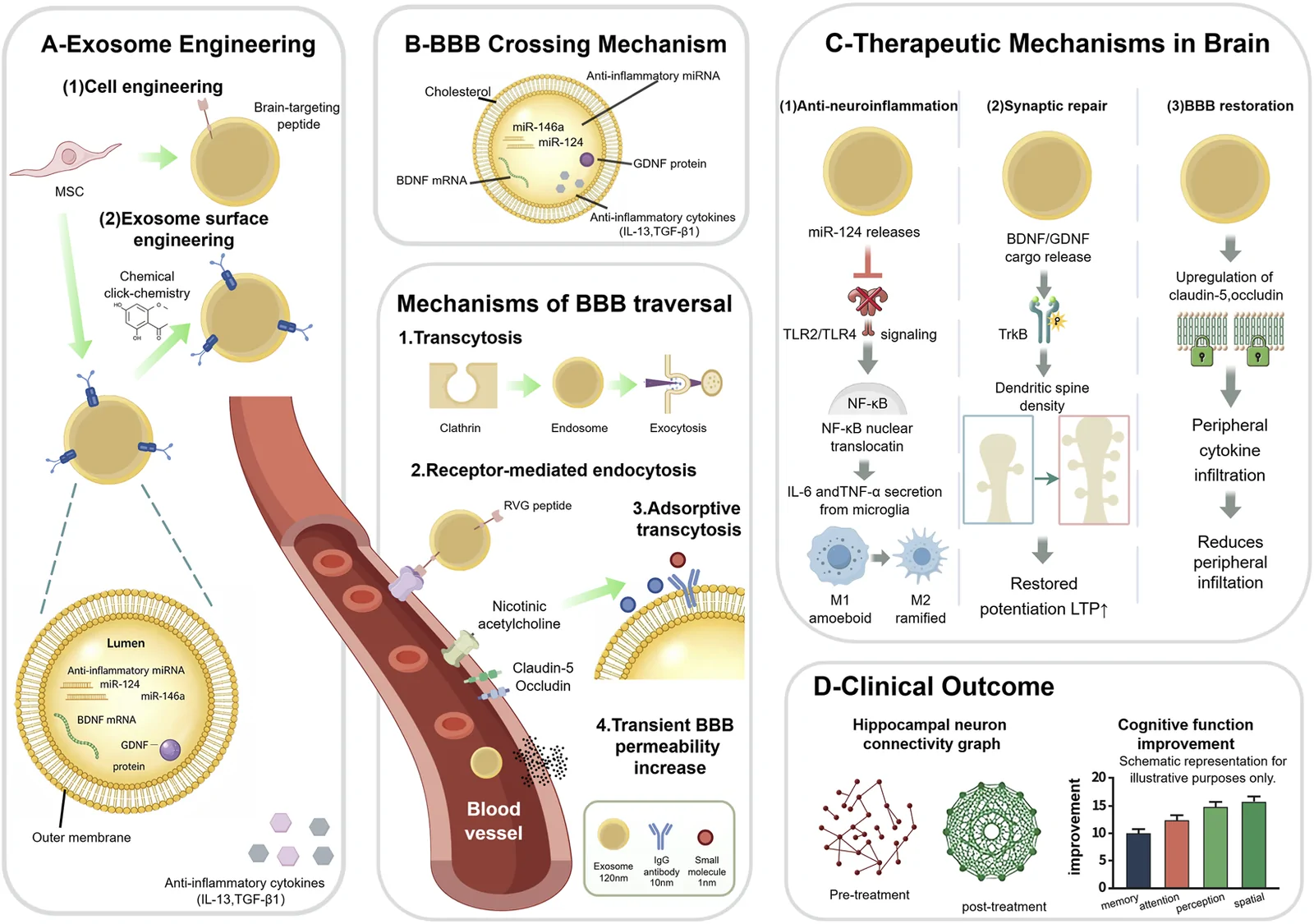
Engineered exosome-based therapeutic strategies for POD and POCD. **(A)** Exosome engineering approaches comprising two strategies:cell engineering, in which MSCs are modified to display brain-targeting peptides on the exosomal surface; and exosome surface engineering via chemical click-chemistry modification. The engineered exosome lumen carries multi-target therapeutic cargo including anti-inflammatory miRNAs(miR-124, miR-146a), BDNF mRNA, GDNF protein, and anti-inflammatory cytokines (IL-13, TGF-β1). **(B)** Mechanisms by which engineered exosomes traverse the blood-brain barrier, including: (1) transcytosis via clathrin-mediated endosome formation and exocytosis; (2)receptor-mediated endocytosis via RVG peptide binding to nicotinic acetylcholine receptors; (3) adsorptive transcytosis; and (4) transient increases in BBB permeability. Size comparison shows exosomes (∼120 nm) relative to IgG antibody (∼10 nm) and small molecules (∼1 nm). **(C)** Therapeutic mechanisms in the brain encompassing three complementary pathways: (1)anti-neuroinflammation, in which miR-124 suppresses TLR2/TLR4 signalling, inhibits NF-κB nuclear translocation, and reduces IL-6 and TNF-α secretion from microglia, promoting M1 amoeboid to M2 ramified polarization; (2) synaptic repair, in which BDNF/GDNF cargo release activates TrkB receptors, increases dendritic spine density, and restores long-term potentiation (LTP↑); and (3) BBB restoration, in which upregulation of claudin-5 and occludin reduces peripheral cytokine infiltration. **(D)** Schematic representation of anticipated clinical outcomes, including improved hippocampal neuron connectivity and enhanced cognitive function across memory, attention, perception, and spatial domains (illustrative purposes only). Abbreviations:MSC, mesenchymal stem cell; BBB, blood-brain barrier; RVG, rabies virus glycoprotein; LTP, long-term potentiation; BDNF, brain-derived neurotrophic factor; GDNF, glial cell line-derived neurotrophic factor; TGF-β1, transforming growth factor-β1; NF-κB, nuclear factor kappa B; TLR, Toll-like receptor. Created with Figdraw.

Beyond their diagnostic applications, exosomes hold considerable therapeutic potential for POD and POCD, owing to their excellent biocompatibility, low immunogenicity,and innate capacity to traverse the blood-brain barrier (Wiklander et al., 2019). Exosomes mediate therapeutic effects primarily through two complementary strategies:as vehicles for targeted drug delivery and as endogenous neuroprotective agents. The engineering strategies, BBB-crossing mechanisms, and downstream therapeutic effects of exosome-based interventions are comprehensively illustrated in Figure 2. It should be acknowledged that exosome-based therapeutic research directly targeting POD and POCD models remains in its early stages, with a relatively limited body of published evidence currently available. Accordingly, this section will prioritize research evidence directly validated in POD and POCD models, while also systematically reviewing the broader and more established research foundation of exosomes in the field of neuroprotection, which collectively provide important mechanistic insights into the biological functions and therapeutic promise of these vesicles.

### 3.1 Exosomes as drug delivery vectors for targeted therapy

The BBB represents the primary obstacle to drug delivery within the central nervous system, as it prevents the vast majority of therapeutic molecules from effectively penetrating brain parenchyma and reaching their intended targets. As naturally occurring endogenous nanovesicles, exosomes can traverse this barrier through multiple mechanisms, including transcellular transport, receptor-mediated intercellular interactions, adsorption-mediated transcytosis, and transient increases in BBB permeability, collectively rendering them ideal drug delivery carriers (Zhang X. et al., 2025). However, native exosomes are subject to several inherent limitations, including weak targeting capacity that relies predominantly on passive mechanisms or limited natural homing, low drug-loading efficiency, restricted yield, and potential batch-to-batch variability (van den Boorn et al., 2013). To address these shortcomings, researchers have developed engineered exosomes that offer advantages including active targeting, enhanced therapeutic potency, and multifunctional integration (Armstrong et al., 2017). Engineered exosomes broadly encompass two categories:cellular engineering approaches performed at the level of the parental cell, and post-isolation exosome engineering methods involving direct modification of purified vesicles (Wang et al., 2021). Through exosome engineering, ligand-functionalized targeted exosomes can be generated for *in vivo* drug delivery. Targeting functionality may be conferred through several strategies:transfecting donor cells with plasmids or viral vectors to express ligand-fusion proteins, such that secreted exosomes naturally display the targeting molecule; surface modification via bioconjugation or lipid insertion techniques; or genetic integration of targeting peptides or proteins into membrane-bound proteins through fusion expression of transmembrane domains, thereby enabling active tissue-specific targeting (Mondal et al., 2023). In the context of POD and POCD, principal therapeutic targets include neuroinflammation, neurotoxic protein aggregation and tau pathology, oxidative stress, and mitochondrial dysfunction. Loading therapeutics into exosomes enables their targeted delivery to the brain, underscoring the need to develop carrier systems capable of precisely delivering anti-inflammatory agents to activated microglia or specific brain regions such as the hippocampus, with the dual aim of enhancing therapeutic efficacy and reducing systemic adverse effects. Among naturally derived exosome sources, MSCs have attracted considerable attention by virtue of their relative ease of procurement, capacity for *in vitro* expansion, and potential to circumvent immune rejection in autologous transplantation settings (Uccelli et al., 2008). MSCs express a broad repertoire of adhesion molecules on their surface, analogous to immune cells, and can migrate directionally to sites of tissue damage or inflammation in response to the chemotactic signals of cytokines such as TGF-β1 and IL-8 (Barcellos-de-Souza et al., 2016; Loebinger et al., 2009). This intrinsic migratory behavior endows MSC-derived exosomes with inherent homing potential that may facilitate their preferential accumulation in brain regions affected by neuroinflammation and injury, rendering them particularly well suited for the treatment of POD and POCD. In a representative study, Wei et al. co-incubated exosomes secreted by bone marrow-derived MSCs with doxorubicin hydrochloride to generate doxorubicin-loaded exosomes (Exo-Dox) (Wei et al., 2019). Exo-Dox exhibited a larger vesicular volume than unmodified exosomes, which was associated with enhanced targeting efficiency. Compared to free doxorubicin, Exo-Dox demonstrated superior cellular uptake and antitumor efficacy in the osteosarcoma cell line MG63 without significant toxicity to normal cells. By combining the tumor-homing properties of MSCs with the tissue infiltration capacity of exosomes, this approach exemplifies how precise targeted delivery of small-molecule chemotherapeutic agents may be achieved. This study demonstrates the feasibility of exosomes as a versatile drug delivery platform; however, their therapeutic efficacy in the specific context of POD and POCD has yet to be validated. Future investigations employing POD/POCD models will be necessary to systematically evaluate the effects of exosome-based drug delivery on neuroinflammation and cognitive function.

### 3.2 The endogenous neuroprotective effects of exosomes

Neuroprotection is broadly defined as the preservation of the structural and functional integrity of injured neurons and their surrounding tissue by mitigating specific events in secondary injury cascades, thereby reducing the incidence and severity of neurological damage. Exosomes derived from glial cells, including astrocytes, microglia, and oligodendrocytes, can modulate intercellular communication within the brain and exert either neuroprotective or neurotoxic effects on neurons, with the directionality of these effects being highly dependent on the activation state of the source glial cells and the prevailing pathophysiological environment. In neuronal cultures, microglia-derived exosomes have been observed to exert protective effects through anti-inflammatory responses and the regulation of synaptic activity. When glial cells are in a homeostatic or benignly activated state, the exosomes they release primarily serve to support and protect neurons; conversely, when glial cells undergo excessive or pathological activation in the context of chronic disease or acute severe injury, the exosomes they release transition into mediators of neuronal damage (Chen et al., 2023). Exosomes occupy a central and dualistic role in neuroprotection. On one hand, exosomes released by homeostatic or benignly activated glial cells directly support neuronal survival and suppress injury by delivering neurotrophic factors, anti-inflammatory mediators, and facilitating the clearance of toxic proteins (Oyarce et al., 2022). On the other hand, they function as key mediators of intercellular signal transduction, coordinating protective communication among cells in the injured microenvironment to promote neural repair and regeneration (Pei et al., 2019). Exosomes derived from MSCs or astrocytes naturally carry a diverse repertoire of neuroprotective and reparative factors, and can directly repair damaged synapses and enhance synaptic plasticity through the delivery of bioactive cargo (Lopez-Leal and Court, 2016; Xin et al., 2013). Concurrently, these vesicles can upregulate the expression of tight junction proteins such as occludin and claudin-5, effectively restoring blood-brain barrier integrity and thereby collectively attenuating the pathological processes associated with POD and POCD (Jeong et al., 2021).

Exosomes derived from certain cell types are capable of exerting neuroprotective effects directly through their intrinsic bioactive constituents, without the requirement for exogenous drug loading. Induced pluripotent stem cell-derived MSC exosomes (iPSC-MSC-Exos) have been shown to improve cognitive function in a diabetic mouse model of POCD by reducing neuroinflammation and promoting neurogenesis (Lang et al., 2023). Antler-derived mesenchymal stem cell exosomes ameliorated cardiopulmonary bypass-induced POCD through inhibition of the TLR2/TLR4 signaling pathway (Yang et al., 2020). In a dual-inflammation mouse model of POCD, Qin et al. reported that macrophage-derived exosomes promote both peripheral and central inflammatory responses, exacerbating blood-brain barrier disruption and cognitive impairment (Qin et al., 2024). In a complementary line of investigation, a study employing an *in vitro* three-dimensional culture model of rat cavernous nerves directly demonstrated that the addition of exosomes derived from adipose-derived mesenchymal stem cells significantly increased both the growth length and axonal density of cavernous nerves (Niu, 2021). Notably, these exosomes were found to contain cytoskeletal components including actin and β-tubulin, which may directly supply structural raw materials to support nerve regeneration. Although studies directly examining exosome-based therapy in POD and POCD models remain relatively limited, a number of investigations conducted in related neurological disease models provide compelling evidence of the neuroprotective potential of exosomes and offer a valuable mechanistic foundation for their application in the perioperative neurocognitive setting. Mesenchymal stem cell-derived exosomes carry an abundance of neurotrophic factors, including GDNF and NGF, as well as anti-inflammatory cytokines such as TGF-β1 and IL-13 (Otero-Ortega et al., 2018). While suppressing neuroinflammation, these vesicles simultaneously deliver survival signals to neurons and oligodendrocyte precursor cells, thereby promoting axonal sprouting and myelin regeneration. MSCs can also modulate cytokine production at the site of injury and reshape the local inflammatory microenvironment. Umbilical cord blood-derived MSCs, for instance, have been shown to promote M2 macrophage polarization, reducing the expression of IL-7,IFN-γ, and TNF-α while concurrently increasing the expression of IL-4, IL-13, and IL-1β (Pang et al., 2022; Bao et al., 2018). A basic research study published in 2024 further demonstrated that MSCs can improve cognitive function in mice. Given that hippocampal neuronal loss and impaired neurogenesis represent hallmark features of POCD, the capacity of MSC-derived exosomes to promote new neuron generation is of particular therapeutic relevance. This effect has been attributed to the concurrent downregulation of pro-inflammatory mediators, including TNF-α, NF-κB, and Iba1, and the upregulation of neurogenesis-associated factors such as DCX and NeuN (Fallahi et al., 2024). In addition to their pro-neurogenic effects, exosomes can repair BBB integrity by upregulating the expression of tight junction proteins, including occludin and claudin-5, in endothelial cells. MSC-derived exosomes have been specifically shown to significantly increase occludin expression at the BBB in POCD model mice, reduce vascular permeability, and limit the infiltration of peripheral inflammatory factors into the brain parenchyma (Chai et al., 2024). Collectively, a growing body of evidence supports the considerable therapeutic promise of MSC-secreted exosomes for the treatment of POCD. It should be emphasized that the studies cited above are derived exclusively from non-surgical neurological injury models, and the findings cannot be directly extrapolated to the perioperative context. The distinct pathophysiological milieu engendered by surgical stress and anesthetic exposure in POD and POCD necessitates dedicated validation through specifically designed animal experimental paradigms.

Astrocyte-derived exosomes likewise possess significant therapeutic value. As key glial cells of the central nervous system, astrocytes play an indispensable role in maintaining neurohomeostasis and shaping neuropathological processes through the secretion of exosomes. Neuroinflammation, which constitutes a core pathological process in neurological disorders including POCD, involves the activation of both microglia and astrocytes (Liu et al., 2023). In response to neural injury and inflammation, astrocytes undergo reactive astrogliosis and assume a dualistic role in the neuroinflammatory milieu, releasing both pro-inflammatory and anti-inflammatory mediators. Beyond their immunomodulatory function, astrocytes express immune receptors, contribute to the phagocytosis of harmful substances,and maintain close structural and functional associations with the blood-brain barrier. Astrocyte dysfunction can therefore precipitate blood-brain barrier disruption, which in turn propagates neuroinflammation and cognitive decline (He et al., 2024). Exosomes released by unstimulated astrocytes carry a repertoire of neuroprotective substances, including fibroblast growth factor-2, vascular endothelial growth factor, and apolipoprotein D. In contrast, exosomes secreted by activated astrocytes are enriched with neuroprotective molecules such as heat shock proteins, synapsin-1, specific microRNAs, and glutamate transporters. Well-characterized astrocyte-derived extracellular vesicles prepared under defined culture conditions may therefore hold meaningful therapeutic potential for the treatment of neurodegenerative diseases, and by extension, for perioperative neurocognitive disorders in which analogous pathological processes are operative (Upadhya et al., 2020).

### 3.3 Advantages of exosome-based therapy

Relative to conventional pharmacological treatments for POD and POCD, exosome-based therapies confer several distinctive advantages.

With respect to biocompatibility and immunogenicity, exosomes are more readily internalized by recipient cells through mechanisms such as membrane fusion and endocytosis, owing to their natural membrane protein composition. They exhibit high biocompatibility alongside potentially superior delivery efficiency, and their inherent homing capacity reduces nonspecific biodistribution. As natural cellular products, exosomes are well tolerated by the host, with immunogenicity substantially lower than that of their parent cells, rendering them considerably less likely to elicit immune rejection and thereby ensuring a favorable safety profile (Malekian et al., 2023). In the treatment of central nervous system diseases, for instance, exosomes derived from bone marrow MSCs can be effectively internalized by target cells and deliver therapeutic cargo directly to the lesion site, while mitigating the systemic toxic adverse effects associated with conventional pharmacotherapy. This exceptional biocompatibility further enables exosomes to maintain stability within the circulatory system and resist rapid clearance, thereby prolonging their duration of action.

Regarding targeting capability, off-target effects represent a primary source of adverse reactions in conventional therapeutics. Chemotherapeutic agents, for example, can damage rapidly dividing normal cells in tissues such as the bone marrow and gastrointestinal tract, while siRNA accumulation in the liver may interfere with normal gene function (Ettinger, 2005; Sivak et al., 2015; Janas et al., 2018). Exosomes can be engineered through genetic or chemical modification to selectively target specific cell types central to disease pathogenesis, including neurons and microglia, thereby enhancing therapeutic precision and reducing off-target toxicity (Sarko and McKinney, 2017). As a representative example, exosomes secreted by engineered iPSC-derived MSCs demonstrated neuroprotective and neurogenic effects in a POCD model in diabetic mice, improving cognitive function through localized action that minimized the likelihood of systemic adverse effects (Lang et al., 2023). Beyond cell-type targeting, surface modification of exosomes can enhance their brain-targeting efficiency and capacity to traverse the blood-brain barrier, promoting their accumulation in affected brain regions (Malekian et al., 2023). Furthermore, functionalization of exosomal surfaces with targeting peptides enables selective binding to and uptake by macrophages, facilitating the delivery of anti-inflammatory siRNA targeting key inflammatory mediators such as NF-κB,TNF-α,or IL-1β,and thereby achieving precise gene silencing at sites of inflammation (Kim et al., 2010).

With regard to multi-mechanism action, exosomes serve as key mediators of paracrine signaling, which constitutes one of the core mechanisms underlying cell-based therapies through which cells repair tissues and modulate immunity by secreting a diverse array of bioactive factors. Exosomes naturally carry membrane proteins that convey the identity information of the source cell, together with proteins and nucleic acids that encode instructional information, collectively enabling them to recapitulate cell-to-cell communication (Gnecchi et al., 2016; van Niel et al., 2018). The complex and heterogeneous mixture of bioactive molecules encapsulated within exosomes confers a unique capacity to simultaneously engage multiple pathological pathways, including neuroinflammatory responses, synaptic dysfunction, and blood-brain barrier disruption. Through these multi-target synergistic effects, exosomes offer the prospect of more comprehensive and integrated therapeutic outcomes for POD and POCD, charting new strategic directions for intervention in perioperative neurocognitive disorders. In ischemic stroke models, engineered exosomes have been shown to simultaneously exert anti-inflammatory effects and promote synaptic remodeling. Given that POD and POCD share core pathological mechanisms with stroke, including neuroinflammation and synaptic damage, these findings provide a meaningful theoretical basis for the potential application of engineered exosomes in the treatment of perioperative neurocognitive disorders. Nevertheless, dedicated model validation in POD and POCD settings is currently lacking, and the clinical translational value of these approaches warrants further rigorous investigation.

## 4 Current challenges and future directions

### 4.1 Current technical challenges

Despite the promising outlook, exosome research in the context of POD and POCD remains predominantly at the preclinical stage, and numerous substantial challenges must be addressed before clinical translation can be realized.

With respect to production and standardization, the volume of exosomes naturally secreted by cells is extremely low,necessitating the culture of vast numbers of cells to obtain therapeutically relevant doses and resulting in prohibitively high production costs. Conventional two-dimensional culture flask systems are ill-suited to meet the demands of large-scale manufacturing, and while the transition to three-dimensional bioreactor culture represents a growing trend, culture conditions require careful optimization to prevent unintended alterations in exosome properties and yield. The development of production methods that are simultaneously cost-effective and scalable therefore remains a critical prerequisite for advancing exosome therapy toward clinical application. Compounding this challenge, the field currently lacks universally accepted quality control standards and standard operating procedures, and key attributes including particle size distribution, purity relative to protein contaminants, and biological potency remain inadequately defined. Ensuring the functional consistency and physicochemical reproducibility of exosomes at scale further demands the resolution of numerous technical hurdles, encompassing efficient collection, purification, and storage, all while preserving their biological fidelity relative to naturally secreted vesicles. The fact that different therapeutic applications may require specific engineering modifications or customization of exosomes introduces an additional layer of manufacturing complexity, further increasing both the technical difficulty and overall cost of production. In the specific context of POD and POCD research, the standardization challenges outlined above are further compounded by disease-intrinsic complexities. POD and POCD themselves lack unified biological definitions and diagnostic criteria, and the two conditions differ substantially in their temporal characteristics and pathophysiological mechanisms. POD typically manifests within days of surgery, whereas POCD may persist for weeks to months, and this divergence in time windows means that the timing of exosome sample collection often varies considerably across studies. Such variability may result in the observation of fundamentally different exosome profile changes at different disease stages, severely compromising the validity of longitudinal comparative biomarker analyses. Furthermore, perioperative factors exert a significant independent influence on exosomal cargo composition, introducing additional confounding variables that are difficult to control. These include the type of anesthesia administered, the extent of surgical trauma sustained, and the analgesic regimens employed, all of which can independently induce systemic inflammatory responses and alter the molecular composition of exosomal cargo, thereby substantially complicating the interpretation of study findings.

With regard to biological and characterization challenges, exosomes secreted by different cell types, including stem cells, immune cells, and tumor cells, differ vastly in their molecular composition and functional properties (Yáñez-Mó et al., 2015). Even within a single cell line maintained under identical culture conditions, heterogeneous subpopulations of exosomes varying in size, density, composition, and function are concurrently secreted (Willms et al., 2018). This inherent heterogeneity renders standardized production exceptionally difficult. Current technologies are insufficient to precisely identify which exosome subpopulation is principally responsible for therapeutic efficacy, nor can they reliably ensure that different production batches exhibit fully consistent therapeutic outcomes and safety profiles. This multi-level heterogeneity poses considerable challenges for both mechanistic research and clinical application, and necessitates the development of more refined isolation and characterization strategies capable of resolving functionally distinct exosome populations with greater precision. Beyond biological heterogeneity, technical variability represents another key challenge affecting the reliability of exosome-based biomarker results. Each of the principal isolation methods currently in use carries inherent limitations:ultracentrifugation, the most widely employed approach, may induce exosome aggregation, membrane structural damage, and protein contamination; polymer precipitation offers high recovery rates but is prone to coprecipitation of lipoproteins and plasma proteins; and size-exclusion chromatography yields high-purity preparations but at the cost of relatively low recovery rates. As a consequence of differences in isolation methods, centrifugation parameters, sample processing workflows, and purification standards across laboratories, inconsistent results may be obtained even when investigating the same disease and the same candidate biomarker. Methodological variability in exosome isolation thus directly and substantially undermines the validity of biomarkers and the reproducibility of research findings, a problem that is particularly pronounced in POD and POCD research. NDEs are currently enriched using surface markers such as L1CAM or NCAM; however, accumulating evidence suggests that these molecules are not entirely neurospecific, raising the possibility that non-neural vesicles are co-isolated, thereby reducing the accuracy of brain-derived marker detection (Norman et al., 2021). Whether exosome-based candidate biomarkers can ultimately achieve clinical translation therefore depends not only on their biological relevance but equally on the establishment of standardized isolation procedures, a unified quality control framework, and characterization strategies that conform to the MISEV guidelines. Future research should place greater emphasis on pre-analytical variables encompassing sample collection, storage,and processing, as well as the systematic selection of isolation methods and the standardization of detection platforms, in order to enhance the reproducibility of findings and the capacity for cross-center validation, thereby ensuring the clinical reliability of exosome biomarkers. In the specific context of POD and POCD, the issue of neuron-derived exosome enrichment purity is of particular importance. The vast majority of studies to date have utilized exosomes isolated from peripheral blood, whereas the core pathological changes in POD and POCD originate within the central nervous system. Due to the absence of highly specific techniques for enriching neuron-derived exosomes, and because the signal contributed by brain-derived vesicles is readily diluted and obscured by the far greater abundance of exosomes originating from platelets and immune cells, reliably distinguishing peripheral inflammatory exosomes from brain-derived exosomes remains a formidable analytical challenge. This limitation directly constrains the sensitivity and specificity of exosomal biomarker detection for POD and POCD, and represents one of the core technological bottlenecks in the field that urgently requires innovative solutions.

With respect to clinical translation and regulatory challenges, despite the substantial progress achieved in laboratory-based exosome research, clinical application remains in its early stages. Only a limited number of clinical trials are currently investigating the efficacy and safety of exosome-based therapies, and the absence of large-scale randomized controlled trials represents a major impediment to their widespread adoption in clinical practice. Clinical trials in cancers such as prostate and lung cancer remain at early phases, and the efficacy, safety, and broader applicability of these therapies require further rigorous validation. Key unresolved issues include the determination of optimal dosing regimens and routes of administration, as well as the identification of predictive biomarkers capable of stratifying patients likely to respond to exosome-based treatment. Moreover, the long-term efficacy and potential adverse effects of exosome therapies remain incompletely understood, which is a critical prerequisite for the establishment of standardized treatment protocols (Li et al., 2025). As an emerging class of biologic therapeutics, exosomes currently face the additional challenge of inconsistent definitions and classifications across regulatory agencies in different countries and regions, a situation that may substantially complicate the approval process. Regulatory uncertainty is particularly consequential in the context of hepatic and colorectal cancer treatment, where it may delay both clinical trial progression and market authorization. Beyond regulatory considerations, exosomes carry inherent risks of triggering immune responses or eliciting unintended biological effects, including the potential to promote tumor invasion and metastasis. Basic research has established that tumor-derived exosomes function as oncogenic messengers capable of reshaping the tumor microenvironment and driving disease progression through the delivery of specific proteins, nucleic acids, and other bioactive constituents (Kalluri, 2016). Accordingly, even though exosomes are generally regarded as low in immunogenicity, their therapeutic use necessitates careful consideration of risks associated with long-term accumulation *in vivo*, potential off-target signaling, and the intrinsic pathogenic potential of exosomes originating from pathological cells (Lener et al., 2015). Thorough preclinical safety evaluation is therefore indispensable to ensure patient safety prior to clinical deployment. The design of clinical trials specifically targeting POD and POCD presents several unique and compounding challenges. First, the diagnosis of both conditions continues to rely on behavioral assessments and lacks a gold standard, introducing inherent subjectivity into the definition of clinical endpoints for exosome intervention trials. Second, the substantial heterogeneity of the perioperative patient population significantly increases the difficulty of identifying and controlling for confounding variables, complicating the attribution of observed effects to exosome-based interventions. Compounding these methodological difficulties, there are currently no specific ethical review standards or regulatory guidelines governing exosome intervention studies targeting POD and POCD, which impedes the standardized design and conduct of relevant clinical trials. Third, existing exosome research on POD and POCD relies predominantly on young, healthy animal models, whereas the clinical high-risk population comprises elderly patients with multiple comorbidities whose preoperative brain state differs substantially from that of the experimental animals employed. Current animal models are therefore unable to fully recapitulate the neurobiological complexity of the clinical patient population, which fundamentally limits the predictive utility of exosome biomarker findings and constrains the translational fidelity of basic research to clinical practice.

### 4.2 Future research directions

To accelerate the clinical translation of exosome research in the context of POD and POCD, future investigations should prioritize the following key areas:

A primary priority for future research is the identification of exosomal biomarkers specific to POD and POCD. The majority of current candidate molecules are derived from studies of Alzheimer’s disease or other neurodegenerative disorders, and their specificity for perioperative neurocognitive disorders remains to be established. Future efforts should integrate multi-omics approaches encompassing proteomics, transcriptomics, and single-exosome analysis technologies to systematically screen for exosome molecular profiles capable of reliably distinguishing POD and POCD from normal postoperative recovery. Such profiles would provide the foundation for developing robust risk prediction models applicable to preoperative risk stratification and early postoperative surveillance.

The development and promotion of standardized production and quality control systems represents another critical priority. There is a pressing need to develop gentler, more efficient, and high-throughput isolation and purification technologies to supplant traditional approaches such as ultracentrifugation, which carries risks of exosome structural damage and coprecipitation of contaminants (Raju et al., 2022). Microfluidic technology, by virtue of its high separation efficiency and minimal mechanical disruption to vesicle integrity, is poised to become a mainstream platform in this regard. Beyond technological advancement, the establishment of a comprehensive set of release standards encompassing particle size, concentration, surface marker expression, purity, drug loading capacity, and sterility and endotoxin levels is essential. The production of exosome-based therapeutics must furthermore be incorporated into Good Manufacturing Practice frameworks, with relevant institutions ensuring that the entire workflow spanning cell source selection and culture through to exosome isolation, formulation, and storage adheres to the highest quality standards, thereby guaranteeing product safety and efficacy. Concurrently, the establishment and widespread adoption of consensus characterization guidelines, such as the MISEV guidelines, is indispensable for ensuring data reproducibility and enabling meaningful cross-study comparisons. In the specific context of POD and POCD research, it is recommended that dedicated standard operating procedures for perioperative exosome sample handling be developed within the overarching framework of the MISEV guidelines. Such SOPs should explicitly define blood collection conditions, anticoagulant selection, centrifugation and pre-treatment parameters, and cryopreservation requirements at each designated preoperative and postoperative time point. Rigorous adherence to these standardized protocols will be essential for ensuring the comparability and integrity of data across multicenter clinical studies, and for enabling the cross-institutional validation necessary to advance exosome biomarkers toward clinical implementation.

Future developments in exosome engineering should extend well beyond the use of unmodified natural vesicles, with the overarching goal of transforming exosomes into next-generation smart nanomedicines. To address the challenge of achieving site-specific accumulation within pathological brain regions such as the hippocampus and cortex following blood-brain barrier traversal, gene engineering or chemical surface modification strategies can be employed to display brain-targeting peptides or antibodies directed against neuroinflammatory markers on the exosome surface, thereby significantly enhancing therapeutic precision and reducing systemic off-target effects. Given the complex and multifactorial pathology of POD and POCD, future engineering efforts should prioritize the design of multi-target cargo systems with synergistic therapeutic activity. Such systems could simultaneously carry multiple bioactive constituents, including anti-inflammatory miRNAs such as miR-124 and miR-146a, neurotrophic factor mRNAs encoding BDNF and GDNF, and anti-apoptotic proteins. Among these, miR-124 has been shown to attenuate neuroinflammation in part through suppression of the TLR2/TLR4 signalling axis, a pathway that is also implicated in the pathogenesis of POCD, as evidenced by studies demonstrating cognitive improvement following TLR2/TLR4 inhibition in cardiopulmonary bypass models (Yang et al., 2020). Beyond conventional cargo delivery, engineered exosomes could serve as vehicles for gene-editing tools such as CRISPR-dCas9 to achieve durable transcriptional regulation of inflammation-related genes, or for metabolic modulators designed to alleviate the neuronal energy deficits that contribute to perioperative cognitive impairment. Of particular importance, the design of engineered exosomes for POD and POCD should be guided by the specific time windows and routes of administration that are feasible and clinically meaningful in the perioperative context. The development of targeted exosomes amenable to intravenous administration during or immediately following surgery, capable of reaching the hippocampus and exerting neuroprotective effects prior to the onset of the inflammatory amplification phase, represents the most clinically translatable objective in this field and should be accorded the highest priority in future engineering efforts.

With respect to deeper mechanistic investigation, high-throughput sequencing, proteomics, and lipidomics should be leveraged to comprehensively characterize the miRNA, mRNA, protein, and lipid constituents carried by exosomes of diverse cellular origins and engineering modifications (Sarko and McKinney, 2017). A central objective of such analyses is the identification of key molecular effectors that play dominant roles in the treatment of POD and POCD, including molecules such as MYCBPAP, which regulates synaptic remodeling, and growth factors that promote neurogenesis (Yan et al., 2023). In-depth mechanistic studies should further elucidate how exosomal cargo modulates downstream signaling pathways upon entry into target cells, such as through inhibition of the TLR2/TLR4 signaling axis to attenuate neuroinflammation, or through the regulation of neuronal survival, synaptic plasticity, and neurogenesis (Yang et al., 2020). Such mechanistic insights will not only enable the precise identification of circulating or cerebrospinal fluid-derived exosome signatures capable of predicting high-risk patients for POD and POCD, but will also facilitate the discovery of biomarkers for therapeutic efficacy assessment, thereby supporting targeted treatment strategies and the dynamic monitoring of therapeutic response.

Regarding rigorous clinical validation, the field requires the implementation of large-scale, multicenter, prospective clinical studies to definitively establish the diagnostic and prognostic value of exosome biomarkers. Such studies should enroll patient populations spanning diverse age groups, disease stages, and ethnic backgrounds, and must employ standardized sample collection, processing, and analytical protocols to ensure data reliability and reproducibility. These efforts will enable robust assessment of the sensitivity and specificity of exosome biomarkers across applications including early diagnosis, disease stratification, treatment response prediction, and prognostic evaluation. In parallel, rigorously designed Phase I and II clinical trials are needed to establish the safety and preliminary efficacy of exosome-based therapeutic regimens in surgical patient populations, thereby laying a solid foundation for subsequent Phase III trials and ultimately advancing the clinical translation of exosome-based diagnostic and therapeutic strategies. Unlike chronic neurodegenerative diseases such as Alzheimer’s disease, POD and POCD exhibit distinct time-dependent characteristics that necessitate a longitudinal rather than cross-sectional research paradigm. Future investigations should therefore focus on delineating the dynamic trajectories of perioperative exosomes by implementing systematic serial sampling before surgery, during the early postoperative period, and at long-term follow-up time points. This approach will enable clarification of the temporal roles of exosomes in the initiation of neuroinflammation, blood-brain barrier disruption, and cognitive recovery, and will facilitate the identification of optimal intervention windows for therapeutic targeting. Building on this foundation, future studies should prioritize the enrollment of high-risk surgical populations and establish predefined longitudinal sampling schedules anchored to preoperative baseline measurements and systematic postoperative follow-ups. The resulting trajectories of exosome biomarker changes should be integrated with standardized neurocognitive assessments and neuroimaging data to enable comprehensive and rigorous validation of their utility for early risk stratification and dynamic disease monitoring.

Future therapeutic strategies for POD and POCD are likely to move beyond single-modality approaches toward multimodal integrated frameworks capable of achieving synergistic effects that exceed the sum of their individual components. The combination of exosomes with cognitive training represents one such promising strategy, wherein exosomes provide the biological substrate for neural repair while cognitive training simultaneously enhances neural network function, thereby establishing a complementary biological-behavioral synergy. Exosomes may also be combined with conventional pharmacotherapy by serving as drug delivery carriers loaded with established neuroprotective agents or anti-inflammatory drugs, thereby improving cerebral bioavailability and targeting precision, enhancing therapeutic efficacy, and reducing systemic adverse effects. Furthermore, exosomes hold potential as a complement to mesenchymal stem cell therapy, either as standalone acellular therapeutic agents or as mediators that amplify the reparative effects of transplanted MSCs through paracrine exosome secretion. Indeed, MSC-derived exosomes are increasingly recognized as one of the core mechanisms underlying the therapeutic activity of MSC-based interventions, and represent a compelling cell-free alternative with favorable safety and scalability profiles. The convergence of these modalities into multimodal integrated therapeutic regimens holds considerable promise for achieving a synergistic effect in the management of POD and POCD, and warrants dedicated investigation in future clinical research.

### 4.3 Clinical translation from the laboratory to the perioperative setting

Although exosomes demonstrate considerable potential as both biomarkers and therapeutic carriers for POD and POCD, their full integration into routine perioperative clinical practice continues to face substantial practical challenges. The perioperative clinical workflow can be broadly divided into three phases:preoperative, intraoperative, and postoperative. The application of exosome biomarkers spans all three phases, with primary relevance to preoperative risk assessment, early postoperative monitoring, and prognostic evaluation. This section will examine the prospective utility of exosomes in preoperative risk prediction and postoperative monitoring, address considerations of practical clinical feasibility, and situate exosome biomarkers within the broader landscape of existing perioperative biomarkers through direct comparison.

#### 4.3.1 Preoperative risk prediction

The occurrence of POD and POCD is closely associated with preoperative neuroinflammatory states, subclinical signs of neurodegenerative disease, and a vulnerable intracerebral environment. Studies have demonstrated that certain preoperative biomolecules are associated with the early prediction of POD and POCD, although the specific contribution of exosome-derived markers to preoperative risk stratification requires further validation. Given their capacity to stably traverse the blood-brain barrier and partition into peripheral biofluids, exosomes offer a means of detecting central nervous system-derived molecular information in peripheral blood and cerebrospinal fluid. In support of this concept, numerous studies have established that neurodegenerative disease-associated biomarkers are detectable in preoperative biological samples. For instance, POD patients have been shown to exhibit significantly elevated levels of T-tau and P-tau proteins in preoperative cerebrospinal fluid, alongside reduced levels of Aβ42, a peptide with recognized neuroprotective properties (Wu et al., 2023). Similarly, elevated preoperative blood levels of VCAM-1 have been associated with a reduced risk of POCD at 3 months postoperatively (Moazzen et al., 2024). On this basis, it is reasonable to hypothesize that exosomal cargo constituents may serve analogously as indicators of pathological brain states and be reliably detected in peripheral blood or other biofluids. Preoperative profiling of exosome-associated neurodegenerative or neuroinflammatory markers, used in conjunction with established clinical assessment scales, could substantially augment the identification of high-risk patients whose brains are already in a subclinical state of injury and are consequently more vulnerable to the cognitive impact of surgical intervention. However, direct experimental evidence in support of this framework remains limited, and larger-scale prospective cohort studies will be essential to validate the preoperative predictive value of exosome biomarkers.

#### 4.3.2 Postoperative monitoring

With regard to postoperative monitoring, serial detection of specific exosomal biomarkers at defined postoperative time points holds promise for the early identification of POD and POCD. Fan et al. reported that plasma miR- -3p levels were significantly elevated in patients who subsequently developed POCD relative to cognitively intact controls, as measured both 1 day preoperatively and 7 days postoperatively, with a diagnostic accuracy of AUC 0.938 that substantially exceeded that of conventional clinical risk factors, providing compelling molecular evidence for the early diagnosis of POCD (Fan et al., 2022). Yan et al. further demonstrated that in patients with POD, the expression of multiple proteins associated with neuroinflammation, vascular permeability, and cellular damage, including Claudin-5, MMP9, S100 B, and TLR2, was significantly upregulated in postoperative plasma exosomes relative to healthy controls (Yan et al., 2024). Although this study did not directly report diagnostic sensitivity and specificity, it identifies candidate molecular targets for the development of a POD diagnostic panel based on exosomal protein profiling. By virtue of their unique biological properties, exosomes may thus serve as real-time indicators of acute neuroinflammatory responses in the postoperative setting. Concerning postoperative treatment evaluation, dedicated clinical studies specifically employing exosome biomarkers to assess the efficacy of POD and POCD interventions remain insufficient; however,a well-founded theoretical rationale and preliminary translational potential exist for their application in the dynamic monitoring of treatment responses. For interventions designed to attenuate neuroinflammation or preserve blood-brain barrier integrity, a progressive decline or normalization of relevant exosomal biomarker levels would be expected to accompany a successful therapeutic response. In support of this framework, studies in POCD animal models have demonstrated that activation of the α7 nicotinic acetylcholine receptor improves cognitive function and downregulates inflammatory mediators through enhancement of the cholinergic anti-inflammatory pathway, suggesting that monitoring inflammation-related exosomal markers could provide a biologically meaningful readout of therapeutic efficacy for analogous interventions in humans (Gong, 2020). Future large-scale clinical trials will nonetheless be required to establish whether dynamic changes in POD and POCD-associated exosomal biomarkers can serve as reliable surrogate endpoints for cognitive improvement.

#### 4.3.3 Practical feasibility

The diagnosis of POD and POCD currently relies predominantly on clinical assessment and neuropsychological testing, modalities that are inherently limited by subjectivity and delayed detection. The identification of highly specific and sensitive biomarkers is therefore critical for enabling timely prediction, diagnosis, and therapeutic intervention. The search for such biomarkers remains in its early stages. Although prior studies have explored the relationship between differential circRNA expression in serum exosomes and POCD following coronary artery bypass grafting, systematic exosomal proteomic and non-coding RNAomic investigations specifically targeting POD and POCD remain severely lacking. Furthermore, while POD and POCD share overlapping pathological pathways such as neuroinflammation and dysregulated cortisol signaling, they also exhibit distinct biomarker profiles, and the specific patterns of exosomal cargo alterations across these two conditions remain poorly characterized, complicating the development of condition-specific diagnostic markers. Methodological inconsistencies in exosome isolation, purification, and characterization represent an additional obstacle, as rapid, standardized, and high-throughput workflows suitable for routine clinical laboratory implementation are currently unavailable. The MISEV guidelines, most recently updated as MISEV2023, represent an important international effort to promote standardization; however, the technical gap between laboratory practice and clinical translation remains substantial. Exosome biomarker detection furthermore typically depends on technically demanding and time-consuming analytical procedures, making it difficult to satisfy the rapidity and cost-effectiveness requirements of clinical diagnostics. Finally, the regulatory pathway from biomarker discovery to approval by the FDA or equivalent agencies demands rigorous sequential validation of analytical validity, clinical validity, and practical utility, representing a formidable translational barrier.

In summary, while exosomes demonstrate considerable theoretical promise and meaningful long-term application prospects as biomarkers for POD and POCD, their near-term clinical translation remains practically challenging, and the field currently occupies a transitional phase between basic research and early translational exploration. Achieving clinical translation will require a concerted and coordinated effort encompassing several strategic priorities:adherence to international guidelines such as MISEV to establish standardized end-to-end protocols spanning sample collection, exosome isolation, and biomarker detection; the design and execution of multicenter prospective cohort studies to systematically screen and validate the diagnostic and predictive utility of exosome-derived biomarkers including proteins, miRNAs, and circRNAs in POD and POCD; and the transformation of complex laboratory techniques into automated, rapid,and cost-effective assay platforms amenable to clinical deployment. Exosome biomarkers thus offer genuine promise for the precision management of POD and POCD, but the journey from laboratory discovery to clinical bedside application remains long, demanding sustained interdisciplinary collaboration, continued investment, and rigorous multilevel validation.

#### 4.3.4 Comparison with existing traditional perioperative biomarkers

The table below summarizes a comparative overview of exosome-derived candidate biomarkers and conventional biomarkers in the context of POD and POCD.

At present, traditional biomarkers remain dominant in the diagnosis and risk prediction of POD and POCD, with several having demonstrated established clinical utility. Exosome-derived candidate biomarkers, by contrast, represent a highly promising emerging direction, with their core advantage residing in their potential to provide earlier, more specific, and mechanistically informative assessments of brain status that conventional biomarkers are unable to offer. Nevertheless, the translation of exosomal biomarkers from laboratory research to clinical practice continues to face critical challenges, encompassing technical standardization, large-scale prospective clinical validation, and cost containment. While the routine clinical application of exosome biomarkers in POD and POCD remains a goal yet to be realized, sustained technological innovation is expected to yield meaningful breakthroughs within the next decade.A comparative overview of exosome-derived candidate biomarkers and conventional perioperative biomarkers is presented in Table 2.

**TABLE 2:** Comparison with existing perioperative biomarkers.

Biomarker category	Representative markers	Advantages	Limitations	Overall appraisal
Inflammatory biomarkers	CRP IL-6 IL-1β TNF-α HMGB1 NLR	1. Technically accessible,low cost,and minimal analytical requirements; well suited to routine clinical use 2. Closely aligned with the core pathological mechanisms of POD and POCD. 3. Certain markers (e.g., IL-6, CRP) have demonstrated utility in preoperative risk prediction	1. Poor specificity:reactive to any surgical trauma, infection,tissue ischemia,or systemic inflammatory state 2. Insufficient diagnostic and predictive accuracy when used in isolation 3. More appropriately regarded as indicators of generalized inflammatory risk rather than precise indices of specific intracranial pathological changes	Primary value lies in suitability for large-scale screening and as a component of multimodal assessment; low specificity and limited independent diagnostic efficacy constrain clinical translation
Neuronal injury biomarkers	NfL S100β GFAP	1. Originate directly from neurons, axons,or glial cells; reflect structural brain injury more proximally than systemic markers 2. Closely associated with core pathological mechanisms of POD and POCD. 3. Several markers (e.g., NfL, GFAP, S100B) have demonstrated predictive value for perioperative neurocognitive risk	1. Certain markers (e.g., S100B) are expressed outside the CNS,compromising neurological specificity 2. Most markers have not achieved clinical standardization or widespread adoption 3. Better suited to indicating neuropathological risk than to identifying the aetiology or anatomical localization of injury 4. Circulating levels are influenced by patient-intrinsic factors including age, renal function, and educational attainment 5. Release kinetics, half-lives, and temporal relationship to clinical symptom onset are complex (e.g., S100B requires measurement within a specific post-injury time window) 6. Clinical utility in mild or subclinical injury remains to be validated	Greater CNS specificity and more direct mechanistic relationship to neuropathological processes; clinical translation hampered by assay standardization challenges and residual non-specificity of certain markers
Conventional neuroimaging	MRI DTI PET	1. Directly visualizes region-specific cerebral changes and provides objective evidence of structural abnormalities 2. Enables assessment of functional connectivity within brain networks 3. Established modalities (e.g., MRI) benefit from high clinical availability and relatively standardized acquisition protocols	1. High cost of advanced imaging technology 2. Practical difficulties of dynamic repeated monitoring preclude use in large-scale screening. 3. Insufficiently sensitive to early subclinical molecular changes preceding macrostructural alterations	Preoperative structural abnormalities identified by neuroimaging are established risk factors for POD/POCD and support preoperative risk stratification; real-time perioperative monitoring and objective therapeutic efficacy evaluation remain practically challenging

## 5 Conclusion

Postoperative delirium and postoperative cognitive dysfunction are serious complications that profoundly impair patient recovery and long-term quality of life. Beyond the immense individual suffering they cause, these conditions impose substantial burdens on families and consume considerable resources within the healthcare system. For decades, the prevention and management of POD and POCD in the perioperative setting have been severely constrained by the absence of specific biomarkers for early identification and the lack of effective targeted therapeutic interventions.

Exosomes, as naturally occurring intercellular messengers and therapeutic carriers, offer a solution of transformative potential to address this unmet clinical need. By transporting central nervous system-specific molecules into the peripheral circulation, they provide a novel and non-invasive source of biomarkers for the early detection and dynamic monitoring of POD and POCD. Furthermore, their exceptional biocompatibility and intrinsic capacity to traverse the blood-brain barrier position them as ideal vehicles for targeted drug delivery and endogenous neuroprotection, and they are expected to assume an increasingly important role in therapeutic applications in the years ahead.

Nevertheless, the translation of exosomes from promising laboratory discoveries into routine clinical practice remains fraught with substantial challenges, encompassing the scale-up and scalability of exosome production, associated manufacturing and technical hurdles, the inherent biological complexity of these vesicles, barriers to clinical translation, regulatory uncertainty, and treatment-related safety considerations. Through sustained technological innovation, the establishment of standardized production and quality control frameworks, advances in precision engineering, deeper mechanistic understanding of perioperative neuropathology, rigorous multilevel clinical validation, and the development of multimodal therapeutic strategies, the full clinical application potential of exosomes can be progressively realized. Looking ahead, exosome-based diagnostic platforms and therapeutic regimens are poised to become an integral component of perioperative medicine, making a meaningful and lasting contribution to the improvement of neurological outcomes and long-term health in surgical patients.
